# The predictive validity of Bayley Scales of Infant and Toddler Development-III at 2 years for later general abilities: Findings from a rural, disadvantaged cohort in Pakistan

**DOI:** 10.1371/journal.pgph.0001485

**Published:** 2023-01-12

**Authors:** Muneera A. Rasheed, Ingrid Kvestad, Fariha Shaheen, Uzma Memon, Tor A. Strand

**Affiliations:** 1 Department of Global Public Health and Primary Care, Centre for International Health, University of Bergen, Bergen, Norway; 2 Regional Centre for Child Mental Health and Welfare, NORCE Norwegian Research Centre, Bergen, Norway; 3 Innlandet Hospital Trust, Lillehammer, Norway; 4 Department of Paediatrics & Child Health, The Aga Khan University, Karachi, Pakistan; 5 People’s Primary Healthcare Initiative, Hyderabad, Pakistan; National University of Sciences and Technology, PAKISTAN

## Abstract

Using data from a rural cohort in Pakistan (N = 1298), the study examined the predictive validity of the Bayley Scales of Infant and Toddler Development (BSID) 3^rd^ edition on later tests of general abilities. The BSID III subscales (cognitive, language and motor) were administered at 2 years; general ability was assessed using the Verbal, Performance and Full-Scale score from the Wechsler Preschool and Primary Scale of Intelligence (WPPSI) III at 4 years, and the Fluid Reasoning Index (FRI) from the Wechsler Intelligence Scale for Children (WISC) V at 8 years. The combined BSID subscales explained 15% of the variation of the WPPSI III full scale, 16% of the Verbal scale, 7% of the Performance scale and 1% of the FRI. BSID III scores at 24 months should be used with caution to predict future intellectual abilities.

## Introduction

Interventions targeting early childhood development (ECD) outcomes have been increasingly recommended to be integrated into existing child health and nutrition programmes to promote development of at-risk children in low-and middle-income countries (LMIC) [1–3]. The aim of such interventions is to benefit brain development in the critical early years of life with the potential consequences for school achievement and adult productivity [4–6]. To evaluate the effect of these ECD interventions on brain development, there are a wide range of measures of ECD available, but the need for culturally sensitive tools for use in LMIC has been highlighted by researchers [7]. The Bayley Scales of Infant and Toddler Development (BSID) [8], developed in the USA, is one of the most frequently used tests to evaluate outcomes for young children under three years and are by many considered the gold standard for the measurement of ECD [9]. Studies from different LMIC settings such as Nepal [10,11], Ethiopia [12], and Iran [13] have indicated psychometric acceptability and reliability of the BSID in these cultural settings. A study from Kenya demonstrated convergent validity with stunting and parental concerns for delay [14]. A multi-site study using data from seven LMIC across Asia, Africa and South American sites found the BSID III scores at 2 years to be valid for research purposes [15].

Given ECD interventions are implemented to affect children’s development in later life, having tools that can inform later function or performance are extremely valuable. This psychometric property of the tool is called predictive validity [16]. Though being highly relevant, evidence from studies examining the predictive validity of the BSID in LMIC is scarce. Two studies with BSID II from Indonesia [17] and Bangladesh [18] before 18 months indicated modest correlations with later abilities. A recent study in Nepal examining the relationship between the BSID III and intelligence scores at age 4, found that the Bayley scores at 6–11, 18–23 and 30–35 months explained 3%, 20% and 36% of the variation of the Wechsler Preschool and Primary Scale of Intelligence when the children were approximately 4 years old [19]. However, studies examining long-term predictive validity during school years are urgently needed to strengthen the case for ECD interventions using psychometrically adequate measures. The Pakistan Early Development Scale-up trial was an intervention study conducted between 2008–2012 [20] with two long-term follow-up evaluations including measures of general abilities at ages 4 [21] and 8 years. The study used the BSID III to assess ECD at age 2, providing an opportunity to evaluate the predictive validity of the tool for later intellectual abilities in a rural, disadvantaged context.

## Methods

### Ethics statement

The study was approved by the Ethics Review Committee of the Aga Khan University (4421-Ped-ERC-16) and the Institutional Review Board at University of Bergen, Norway (124126). Parents of the children part of the study provided written informed consent and could refuse to participate at any point during the study.

### Setting

The study includes data from longitudinal follow-ups of an intervention cohort conducted in Naushahro Feroze, Sindh, Pakistan [20]. Pakistan has high rates of maternal mortality (140/100,000), under-5 child mortality (67/1000) and stunting (38%) [22]. National level data are not available for the 0–3 age group with respect to learning environment at home or responsive care. Using a composite indicator of under-5 poverty or stunting, data suggests about half of the young children are at risk of poor development [22]. Naushahro Feroze, predominantly a rural district has poor education indicators. High-quality preschool services specifically are almost non-existent [23]. In the original trial, the cohort of children was exposed to either Responsive Stimulation, Enhanced Nutrition, a combination of these, or standard care in the first two years of life starting from birth [20].

### Procedures

At 2 years, data on socioeconomic status (SES), food security, maternal education and child height was taken along with developmental assessment using the BSID III. The BSID III was administered by a team of 8 trained community-based child development assessors (CCDA) at the homes of the children. Height was measured to the nearest 0·1 cm and weight was measured to the nearest 0·1 kg by another team of trained data collectors. SES and food security were assessed using standard questionnaires described in a previous manuscript [20]. The SES score was generated with a principal component analysis after assignment of scores to individual variables, including ownership of property, livestock and access to water and electricity.

The assessments at later ages; at 4 and 8 years were conducted in closed spaces specifically set-up for the assessments within each village. These spaces were arranged by the data assessment team prior to data collection in respective areas. About 4 assessors at age 2 years were also part of the evaluation team at the two later assessments (4 and 8 years). Given the large team of CCDAs, standard protocols were created with robust quality assurance strategies in place (led by the first author) throughout the data collection phases across the three time points. The procedures included training and practice with observation by the supervisor prior to collecting data, periodic refresher trainings (including for the data entry check team), weekly debriefing meeting for team learning and discussion of ongoing issues and interobserver agreement with supervisor for approximately 10% of the data (N = 125). The details of the approach to training and supervision have been described in another manuscript [24].

### Measures

We used the BSID III to assess development at 2 years using three domain scores: Cognitive, Motor and Language. No cultural adaptations were made to BSID III items. The raw scores were converted into composite scores using the US norms with an expected mean of 100 and Standard Deviation (SD) of 15 [9]. IQ at age 4 years was assessed using an adapted version of the WPPSI III (Rasheed et al, 2018). Seven subtests entailing 3 Verbal (Vocabulary, Information, Word Reasoning), 3 Performance (Block Design, Matrix Reasoning, Picture Concepts), and 1 Processing speed (Symbol Search) of the WPPSI III were used. From these subtest scores, we derived a Full-Scale IQ (FSIQ) and a Verbal and Performance IQ (VIQ and PIQ) score using the US norms. At age 7–8 years, we used the Wechsler Intelligence Scale for Children (WISC) V [25]. At this time point, only two subtests (Matrix Reasoning and Figure Weights) which form the Fluid Reasoning Index (FRI) scores were administered due to time constraints. WISC V was part of a large battery being administered to children and it was not feasible to dedicate more time to administering the complete WISC subscales. The interobserver agreement data between the CCDAs and the supervisor for BSID III using the intraclass correlation coefficient (R ranging from .95 to .99, p < .0001) [20] and WPPSI III (R ranging from .94-.99, p < .0001) [21] has been published earlier and was high. For the WISC V, the agreement measured by intraclass correlations was similarly found to be high for both subscales with .999 (p < .00001) for Matrix Reasoning and 1 (p < .00001) for Figure Weights. As with the previous measures, we used the US norms available in the manual for FRI scores.

### Analysis

The analysis was conducted in Stata v16. We computed the Mean (Standard Deviation, SD) and Median (Interquartile range, IQR) scores of the three different assessment measures: BSID III (Cognitive, Motor and Language), WPPSI III (FSIQ, VIQ and PIQ) and WISC V (FRI). Correlations within and between the subscales of the BSID III and WPPSI III were examined. To examine the predictive ability of BSID III for later abilities we used linear regression models. We extracted the R square from single independent variable and multiple regression models with the BSID III subscale composite scores (Cognitive, Motor and Language) as exposures and the WPPSI III FSIQ, VIQ and PIQ and the WISC IV FRI score as outcomes. In the supplementary material, we also present models with other known determinants for general abilities with and without the BSID III composite scores. We also tested the model after stratification by sociodemographic and anthropometric factors which influence ECD outcomes i.e., socioeconomic status, maternal literacy (ability to read and write), child gender and nutritional status. Further, we categorized the BSID III subscale composite scores in three categories: composite score >-1 SD (>85), between -1 and -2 SD (70 to 85) and <-2SD (<70) based on the US normative data available. We compared the mean (SD) WPPSI III and WISC V scores for each BSID III category using scores >-1SD (85) as the reference.

## Results

Table 1 presents the demographic profile of the participants (N = 1298) at 2 years of age. Of these, 46% (599) were females. Maternal literacy was 31.4% and 17% of the mothers had between 1–5 years of education completed. A similarly high number of mothers (36%) were at risk of depression. At 2 years, 61% of the children were stunted, and 43% were underweight indicating that undernutrition was common.

**Table 1:** Sociodemographic characteristics of the cohort at 2 years (N = 1298).

Variable	
Gender (n, %)	
Female	601 (46.3)
SES index (Mean, SD)	-.01 (0.98)
Household food security (n, %)	
Not food insecure	821 (63.3)
Mildly food insecure	171 (13.2)
Moderately food insecure	219 (16.9)
Severely food insecure	87 (6.7)
No. of siblings (Mean, SD)	3.7 (2.3)
Maternal literacy (n, %)	410 (31.6)
Years of education (n, %)	
None	888 (68.4)
1–5	232 (17.9)
6–10	127 (9.8)
10+	51 (3.9)
Mothers at risk of depression	463 (35.7)
Stunted (HAZ<-2SD) (n, %)	787 (60.6)
Moderate-severely stunted	436 (33.6)
Severely stunted	351 (27.1)
Underweight (WAZ<-2SD) (n, %)	561 (43.2)
Moderate-severely underweight	357 (27.6)
Severely underweight	200 (15.6)

Note: Data is presented as N (%) or Mean (SD) as appropriate.

SES = socioeconomic status, HAZ = Height-for-Age Z-score, WAZ = Weight-for-Age Z-score.

The BSID III mean composite scores ranged from 78.24 (SD 14.61) to 88.74 (SD 17.04) with the highest being for the Motor scale (Table 2). The WPPSI III subscales also had similar scores with a mean of 75.56 (SD 7.56) on the Full scale, 77.12 (SD 9.96) on the Verbal and 79.48 (SD 9.36) on the Performance subscale. The mean score on the FRI of WISC V when the children were 7–8 years was 60.45 (SD 10.85).

**Table 2:** Mean (Standard Deviation) and Median (interquartile range) of the scores on the BSID III, WPPSI III and WISC V sub-scales of the cohort.

Scale	N	Mean (SD)	Median (IQR)	Min	Max
BSID Cognitive composite score	1,298	78.2 (14.6)	80 (65, 90)	55	140
BSID Language composite score	1,298	82.8 (13.4)	83 (74, 91)	39	150
BSID Motor composite score	1,298	88.7 (17.0)	91 (76, 100)	46	154
WPPSI Full Scale IQ	1,233	75.6 (7.6)	75 (71, 80)	55	122
WPPSI Verbal IQ	1,233	77.1 (9.8)	75 (72, 81)	53	131
WPPSI Performance IQ	1,233	79.5 (9.1)	79 (73, 84)	55	129
WISC V FRI	1,141	60.9 (10.4)	61 (55, 67)	45	109

BSID = Bayley Scale of Infant Development, FRI = Fluid Reasoning Index, WISC = Wechsler Intelligence Scales for Children, WPPSI = Wechsler Preschool and Primary Scales for Children.

The correlations between BSID subscale scores and WPPSI III IQ scores were moderate and significant ranging from .24 to .37 (Table 3). Among the three BSID subscales, the Language score was the most highly correlated with future WPPSI III scores, with an *r* of .39 (p<0.05) with WPPSI VIQ, *r* of .26 (p<0.05) with PIQ and .38 with FSIQ at 4 years and an r of .12 (p<0.00) with the WISC FRI at 8 years. WISC FRI had very low correlations with the BSID and WPPSI subscales ranging from .05 to .12.

**Table 3:** Correlation coefficients with p-values between the BSID III composite scores, WPPSI III Verbal, Performance and Full-Scale IQ and the WISC V FRI score of the cohort.

	BSID Cognitive score	BSID Language score	BSID Motor score	WPPSI Verbal IQ score	WPPSI Performance IQ score	WPPSI Full Scale IQ score
BSID Language score	.71					
(N = 1298)	<.001					
BSID Motor score	.72	.78				
(N = 1298)	<.001	<.001				
WPPSI Verbal IQ score	.34	.39	.35			
(N = 1233)	<.001	<.001	<.001			
WPPSI Performance IQ score	.25	.26	.25	.47		
(N = 1233)	<.001	<.001	<.001	<.001		
WPPSI Full Scale IQ score	.34	.38	.35	.87	.82	
(N = 1233)	<.001	<.001	<.001	<.001	<.001	
WISC-FRI score	.08	.12	.09	.09	.05	.10
(N = 1141)	.010	<.001	.004	.004	.093	.001

Note: BSID = Bayley Scale of Infant Development, CI = Confidence Interval, FRI = Fluid Reasoning Index, WISC – Wechsler Intelligence Scales for Children.

The BSID III at 24 months explained 15% of the variation of the WPPSI FSIQ, 16% of the VIQ and 8% of the PIQ (Table 4). For the WISC V FRI scores at 7–8 years, the BSID III explained 1% (Table 5). Analysis including anthropometric and sociodemographic variables with BSID III in the model, increased the explained variance with the WPPSI III to 20% and of the WISC FRI to 2% (S1 Table). Notably, models including only anthropometric and sociodemographic variables explained 13% and 0.5% of the variance of the WPPSI III and the WISC FRI, respectively.

**Table 4:** Associations between BSID-III composite and the WPPSI III Full scale, Verbal and Performance IQ scores at 4 years in the cohort (N = 1233).

Full Scale IQ	Single independent variable analysis				Adjusted multiple regression analysis Combined explained (R^2^ = 0.15)		
	coef.	95% CI	p	R^2^	coef.	95% CI	p
BSID Cognitive scale score	.18	.15 .21	<0.001	.12	.08	.04 .12	<0.001
BSID Language scale score	.20	.17 .23	<0.001	.13	.11	.06 .15	<0.001
BSID Motor scale score	.15	.13 .18	<0.001	.12	.04	.01 .08	.022
Verbal IQ					Combined explained (R^2^ = 0.16)		
	coef.	95% CI	p	R^2^	coef.	95% CI	p
BSID Cognitive scale score	.23	.19 .26	<0.001	.11	.08	.04 .14	.001
BSID Language scale score	.27	.24 .31	<0.001	.13	.15	.10 .22	<0.001
BSID Motor scale score	.21	.17 .23	<0.001	.12	.06	.00 .11	.019
Performance IQ s					Combined explained (R^2^ = 0.08)		
	coef.	95% CI	p	R^2^	coef.	95% CI	P
BSID Cognitive scale score	.16	.13 .19	<0.001	.06	.08	.03 .13	.002
BSID Language scale score	.17	.13 .21	<0.001	.06	.07	.01 .13	.014
BSID Motor scale score	.13	.11 .16	<0.001	.06	.04	-.00 .09	.072

Note: BSID = Bayley Scale of Infant Development, CI = Confidence Interval, IQ = Intelligence Quotient, WPPSI = Wechsler Preschool and Primary Scales of Intelligence.

**Table 5:** Associations between BSID III composite and the WISC V FRI scores at 8 years in the cohort (N = 1141).

BSID scale	Single independent variable analysis				Adjusted multiple regression (R^2^ = 0.01)		
	coef.	95% CI	p	R^2^	coef.	95% CI	p
BSID Cognitive scale score	.05	.01 .09	.015	.01	-.00	-.06 .06	.906
BSID Language scale score	.08	.04 .13	<0.001	.01	.08	.01 .15	.023
BSID Motor scale score	.05	.02 .09	.003	.01	.01	-.05 .07	.777

Note: BSID = Bayley Scale of Infant Development, CI = Confidence Interval, FRI = Fluid Reasoning Index, WISC–Wechsler Intelligence Scales for Children.

The mean difference between WPPSI and WISC scores in the BSID reference group (BSID III scores >-1SD (85) and the other two BSID categories (BSID III scores between -1 and -2 SD (70 to 85) and <-2SD (<70) are shown in Tables 5 and S2. The mean differences between the reference group with the other groups across the three BSID III sub-scales scores ranged from -3.45 to -5.28 for the WPPSI FSIQ. The mean differences were lower for the WISC FRI ranging from -1.33 to -4.07 (Table 6). Similar analysis for WPPSI PIQ and VIQ has been presented as a S2 Table.

**Table 6:** Mean (SD) of the FSIQ at 4 years and FRI at 8 years by BSID III composite score at 2 years and the mean difference in the cohort.

		FSIQ (N = 1233)					FRI (N = 1141)				
BSID III subscales		Mean	SD	Mean diff	95% CI		Mean	SD	Mean diff	95% CI	
Cognitive	> -1 SD (>85)	79.26	8.78	Ref			62.11	10.98	Ref		
	-1 to -2 SD (70 to 85)	75.03	6.35	-4.23	-5.44	-3.03	60.78	10.26	-1.33	-3.17	0.50
	< -2 SD (<70)	73.14	6.44	-5.17	-7.32	-4.92	60.25	9.936	-1.85	-3.66	-.06
Language	> -1 SD (>85)	77.94	7.94	Ref			62.28	10.88	Ref		
	-1 to -2 SD (70 to 85)	73.55	6.34	-4.39	-5.43	-3.36	60.29	9.89	-1.99	-3.53	-0.46
	< -2 SD (<70)	72.66	6.85	-5.28	-6.81	-3.75	58.21	9.146	-4.07	-6.3	-1.85
Motor	> -1 SD (>85)	77.21	7.95	Ref			61.98	10.72	Ref		
	-1 to -2 SD (70 to 85)	73.75	6.08	-3.45	-4.57	-2.35	59.68	9.82	-2.29	-3.92	-0.67
	< -2 SD (<70)	72.34	6.88	-4.87	-6.36	-3.38	59.5	9.59	-2.48	-4.61	-0.36

CI = Confidence Intervals. FSIQ = Full Scale IQ, FRI = Fluid Reasoning Index, BSID = Bayley Scales of Infant Development.

The stratified analysis indicated higher predictive validity of BSID III in low-risk categories (not stunted, belonging to higher SES group, born to mothers who can read and write) compared to high-risk categories (S3 and S4 Tables).

## Discussion

We examined the predictive ability of the BSID III at 2 years for intellectual abilities at age 4 years and 7–8 years in 1292 children participating in an intervention cohort in a marginalised context in rural Pakistan. We found that the BSID III subscales explained 15% of the variation in the WPPSI II full scale IQ at 4 years and only 1% of the variation in the FRI from the WISC V when the children were 7–8 years old.

To the best of our knowledge, the current study is one of very few studies measuring the long-term relationship between BSID III in early childhood and general abilities measured in later childhood in a LMIC and is most likely unique in terms of its large sample size. The associations between the BSID and the WPPSI scores at 4 years are similar to what was reported in a study from Nepal where the BSID scores at approximately 2 years explained 20% of the variation [19]. Notably, the predictive ability for the IQ scores at 4 years was lower in high-risk groups compared to the low-risk group. One possible explanation could be negative influences on child development scrambled the relationship between the measures more in the disadvantaged children than those who were better off. In other words, the lower predictive ability in the disadvantaged children is because these are the children who do not reach their developmental potential.

The poor relationship between BSID III and WISC V at later years is not very different than what was reported with BSID II for later intellectual abilities from Bangladesh [17] and Indonesia [18] where only modest correlations were found between scores at 18 months and the later intelligence scores. The poor associations could be due to the fact that we used only one of the indices in the WISC test (i.e., the Fluid Reasoning Index score) reflecting performance and non-verbal skills only and not the Full-Scale IQ score. This also aligns with the variation explained in the Performance Scale (7%) at age 4 which is substantially lower than what was explained in the Verbal Scale (16%). The inclusion of a full WISC assessment generating both a full IQ score and Verbal scores would have made it possible to follow the patterns found at 4 years whereby the Verbal Scale had stronger associations with the BSID. A study in Chile examining cognitive delay in 6–8 years old school children found the BSID III to have a positive predictive value of 24% [26]. The sample was from an urban setting of children attending an ambulatory clinic unlike our study which was from a non-clinical but disadvantaged context. Moreover, we have used the scores of general abilities on a continuous scale, while the study in Chile, conducted in a community sample to elucidate pathways of change for responsive care, used the WISC score dichotomized for cognitive delay. The relatively low mean scores at age 7–8 years (60) compared to the earlier time point at 4 years (75) may also be an indication of the inability of the environment to sustain the benefits of early exposure given the high risks in the environment e.g., low maternal literacy, poor quality of schools and child malnutrition [27].

Among the three domains of BSID III, the language domain was mostly associated with intellectual abilities of both 4- and 8-year-olds. Several studies have indicated the role of early language skills for later cognitive abilities [28–30]. Some studies have found language to be a mediator between home environment, school readiness [31] and academic achievement in school [32]. In a 29-year follow-up of a general US population sample, Schoon et al. (2010) found a significant risk for poor adult literacy among children with early language problems ([33]. These findings suggest, in accordance with our findings, early language skills to be of particular importance in predicting later abilities. These findings should be considered preliminary, however, and needs to be confirmed in future studies.

The study strengths include high-quality longitudinal data collected in a highly disadvantaged context posing multiple risks to ECD, a large community sample size and low attrition across the different time points. A major limitation is the absence of the Verbal scale and the Full-Scale IQ at age 7–8 years. Another limitation is that we only have BSID measures from one time point, while it could be argued that that using multiple time-points is necessary to evaluate developmental trajectories [34]. Furthermore, the predictive validity of BSID administered at different times in the period of early childhood may be different than when administered to 2-year-olds.

In conclusion, BSID III at 24 months has a moderate correlation with general intellectual abilities at age 4 years but is only weakly correlated with FRI as an index of intellectual abilities during early school years (7–8 years) in this Pakistani cohort. BSID III at 2 years should be used with caution to predict later abilities in early intervention studies especially in high-risk environments. An implication would be to explore the unique PEDS Trial cohort that has data available at longitudinal time points for early measures (e.g., vocabulary development) which most strongly can explain later intellectual abilities.

## Supporting information

S1 Table Single variable and adjusted regression analyses of covariates (anthropometric and sociodemographic variables) with and without BSID III composite scores to predict WPPSI III and WISC FRI scores. (DOCX)

S2 Table Mean (SD) of the VIQ at and PIQ at 4 years by BSID III composite score categories at 2 years and the mean difference between scores in children from rural Pakistan (N = 1233). (DOCX)

S3 Table Associations between BSID composite and WPPSI FSIQ scores by risk factor categories. (DOCX)

S4 Table Associations between BSID composite and WISC FRI scores by risk factor categories. (DOCX)
