# Supplementary material for: The predictive validity of Bayley Scales of Infant and Toddler Development-III at 2 years for later general abilities: Findings from a rural, disadvantaged cohort in Pakistan
Source: PLOS Glob Public Health. 2023 Jan 12;3(1):e0001485. doi: 10.1371/journal.pgph.0001485 (PMC10021670; doi:10.1371/journal.pgph.0001485)
Supplement: S1 Table — (DOCX) [file pgph.0001485.s001.docx]

S1 Table

Single variable and adjusted regression analyses of covariates (anthropometric and sociodemographic variables) with and without BSID III composite scores to predict WPPSI III and WISC FRI scores

| Full Scale IQ (N=1233) | Model 1 (covariates only)  Combined explained (R^2^=.13) | | | | Model 2 (covariates+BSID)  Combined explained (R^2^=0.20) | | | | |  |
| --- | --- | --- | --- | --- | --- | --- | --- | --- | --- | --- |
|  | coef. | 95% CI | p |  | coef. | 95% CI | p | |  |  |
| BSID Cognitive scale score | - | - | - |  | .06 | .02 .09 | .002 | |  |  |
| BSID Language scale score | - | - | - |  | .08 | .04 .13 | <0.001 | |  |  |
| BSID Motor scale score | - | - | - |  | .02 | -0.01 .06 | 0.206 | |  |  |
| HAZ score | 1.3 | .06 1.7 | <.0001 |  | .76 | .38 1.14 | <0.001 | |  |  |
| SES | 1.7 | 1.2 2.1 | <.0001 |  | 1.19 | 074 1.64 | <0.001 | |  |  |
| Maternal literacy | -.83 | -.04 1.5 | .082 |  | -.63 | -1.54 .27 | .173 | |  |  |
| Child gender | .76 | 1.2 2.1 | .063 |  | -.53 | -1.30 .24 | .175 | |  |  |
| FRI (N=1141) | Model 1 (covariates only)  Combined explained (R^2^=.005) | | | | Model 2 (covariates+BSID)  Combined explained (R^2^=0.02) | | | | |  |
|  | coef. | 95% CI | p |  | coef. | 95% CI | | p | | |
| BSID Cognitive scale score | - | - | - |  | -.01 | -.06 0.54 | | .859 | | |
| BSID Language scale score | - | - | - |  | .07 | .00 0.13 | | .047 | | |
| BSID Motor scale score | - | - | - |  | .02 | -.04 0.07 | | .596 | | |
| HAZ score | -.17 | -.74 .40 | .563 |  | -.42 | -1.02 0.17 | | .165 | | |
| SES | .84 | .14 1.5 | .019 |  | .58 | -.14 1.31 | | .118 | | |
| Maternal literacy | -.53 | -1.9 .91 | .468 |  | -.46 | -1.91 0.97 | | .527 | | |
| Child gender | -.49 | .14 1.5 | .425 |  | .50 | -.71 1.71 | | .416 | | |

BSID=Bayley Scales of Infant Development, SES=socioeconomic status
