# Supplementary material for: The predictive validity of Bayley Scales of Infant and Toddler Development-III at 2 years for later general abilities: Findings from a rural, disadvantaged cohort in Pakistan
Source: PLOS Glob Public Health. 2023 Jan 12;3(1):e0001485. doi: 10.1371/journal.pgph.0001485 (PMC10021670; doi:10.1371/journal.pgph.0001485)
Supplement: S2 Table — (DOCX) [file pgph.0001485.s002.docx]

S2 Table

Mean (SD) of the VIQ at and PIQ at 4 years by BSID III composite score categories at 2 years and the mean difference

between scores in children from rural Pakistan (N=1233)

|  |  | VIQ | | | | | PIQ | | | | |
| --- | --- | --- | --- | --- | --- | --- | --- | --- | --- | --- | --- |
|  |  | Mean | SD | Mean diff | 95% CI | | Mean | SD | Mean diff | 95% CI | |
| Cognitive | > -1 SD (>85) | 81.89 | 11.05 | Ref |  | | 82.98 | 10.84 | Ref |  | |
|  | -1 to -2 SD (70 to 85) | 76.40 | 8.48 | -5.49 | -6.82 | -4.16 | 79.34 | 8.19 | -3.66 | -4.94 | -2.40 |
|  | < -2 SD (<70) | 74.06 | 8.98 | -7.83 | -9.15 | -6.51 | 77.04 | 8.36 | -5.86 | -7.11 | -4.60 |
| Language | > -1 SD (>85) | 80.55 | 10.08 | Ref |  | | 81.52 | 9.93 | Ref |  | |
|  | -1 to -2 SD (70 to 85) | 74.01 | 8.46 | -6.49 | -7.63 | -5.37 | 78.27 | 8.45 | -3.37 | -4.46 | -2.28 |
|  | < -2 SD (<70) | 73.66 | 9.58 | -6.88 | -8.55 | -5.21 | 76.61 | 8.24 | -5.11 | -6.72 | -3.51 |
| Motor | > -1 SD (>85) | 79.42 | 10.22 | Ref |  | | 81.06 | 9.81 | Ref |  | |
|  | -1 to -2 SD (7 to 85) | 74.53 | 8.22 | -4.85 | -6.07 | -3.63 | 78.33 | 8.46 | -2.82 | -3.97 | -1.67 |
|  | < -2 SD (<70) | 73.00 | 9.72 | -6.34 | -7.97 | -4.71 | 76.22 | 7.87 | -5.08 | -6.61 | -3.55 |

CI=Confidence Intervals, VIQ=Verbal IQ, PIQ=Performance IQ, BSID=Bayley Scales of Infant Development
