# Supplementary material for: The predictive validity of Bayley Scales of Infant and Toddler Development-III at 2 years for later general abilities: Findings from a rural, disadvantaged cohort in Pakistan
Source: PLOS Glob Public Health. 2023 Jan 12;3(1):e0001485. doi: 10.1371/journal.pgph.0001485 (PMC10021670; doi:10.1371/journal.pgph.0001485)
Supplement: S3 Table — (DOCX) [file pgph.0001485.s003.docx]

S3 Table

Associations between BSID composite and WPPSI FSIQ scores by risk factor categories

| Sociodemographic variables | BSID III scales | At-risk category | | | |  | Low risk category | | | |  |
| --- | --- | --- | --- | --- | --- | --- | --- | --- | --- | --- | --- |
|  |  | coef. | 95% CI | p | R^2^ | coef. | | 95% CI | p | R^2^ | |
| Stunting  n=738, 495 | Cognitive scale | .026 | -.019 .072 | .261 | .09 | .135 | | .068 .203 | .000 | .16 | |
|  | Language scale | .091 | .037 .144 | .001 |  | .109 | | .031 .188 | .006 |  |  |
|  | Motor scale | .051 | .004 .098 | .032 |  | .022 | | -.041 .084 | .493 |  |  |
| SES  n=673, 560 | Cognitive scale | .012 | -.039 .064 | .630 | .07 | .111 | | .053 .169 | .000 | .19 | |
|  | Language scale | .032 | -.020 .085 | .229 |  | .196 | | .119 .273 | .000 |  |  |
|  | Motor scale | .083 | .035 .131 | .001 |  | -.011 | | -.071 .048 | .720 |  |  |
| Maternal ability to read and write  n=837, 396 | Cognitive scale | .050 | .004 .096 | .031 | .09 | .111 | | .038 .184 | .003 | .21 | |
|  | Language scale | .059 | .004 .113 | .033 |  | .181 | | .101 .261 | .000 |  |  |
|  | Motor scale | .063 | .019 .107 | .004 |  | -.011 | | -.074 .072 | .097 |  |  |
| Child gender  n=567, 666 | Cognitive scale | .079 | .024 .133 | .004 | .14 | .071 | | .014 .127 | .014 | .15 | |
|  | Language scale | .097 | .033 .160 | .003 |  | .126 | | .060 .191 | .000 |  |  |
|  | Motor scale | .036 | -.018 .091 | .190 |  | .049 | | -.004 .102 | .074 |  |  |

Note: N is presented for at-risk category, low risk category

At risk category for: stunting is <-2SD, SES is below mean SES for the sample, maternal literacy is inability to read and write and child gender is female.
