# Supplementary material for: The predictive validity of Bayley Scales of Infant and Toddler Development-III at 2 years for later general abilities: Findings from a rural, disadvantaged cohort in Pakistan
Source: PLOS Glob Public Health. 2023 Jan 12;3(1):e0001485. doi: 10.1371/journal.pgph.0001485 (PMC10021670; doi:10.1371/journal.pgph.0001485)
Supplement: S4 Table — (DOCX) [file pgph.0001485.s004.docx]

S4 Table

Associations between BSID composite and WISC FRI scores by risk factor categories

| Risk variables | BSID III scales | At-risk category | | | |  | Low risk category | | | | |
| --- | --- | --- | --- | --- | --- | --- | --- | --- | --- | --- | --- |
|  |  | coef. | 95% CI | p | R^2^ | coef. | | 95% CI | p | R^2^ |  |
| Stunting  n=682, 459 | Cognitive scale | -.016 | -.092 .059 | .671 | .01 | .007 | | -.090 .105 | .881 | .00 |  |
|  | Language scale | .064. | -.022 .151 | .146 |  | .960 | | -.015 .207 | .090 |  |  |
|  | Motor scale | .051 | -.026 .127 | .198 |  | -.040 | | -.130 .049 | .380 |  |  |
| SES  n=628, 560 | Cognitive scale | -.023 | -.105 .059 | .058 | .00 | .007 | | -.081 .097 | .863 | .00 |  |
|  | Language scale | .090 | .007 .173 | .032 |  | .047 | | -.069 .164 | .426 |  |  |
|  | Motor scale | .004 | -.071 .079 | .915 |  | .011 | | -.079 .102 | .802 |  |  |
| Maternal ability to read and write  n=362, 779 | Cognitive scale | -.023 | -.091 .044 | .501 | .01 | .026 | | -.095 .148 | .674 | .00 |  |
|  | Language scale | .111 | .031 .191 | .007 |  | .013 | | -.116 .142 | .842 |  |  |
|  | Motor scale | -.009 | -.075 .056 | .75 |  | .038 | | -.079 .156 | .522 |  |  |
| Child gender  n=520, 621 | Cognitive scale | .003 | -.081 .088 | .942 | .00 | -.011 | | -.095 .073 | .798 | .00 |  |
|  | Language scale | .061 | -.035 .158 | .213 |  | .094 | | -.002 .191 | .057 |  |  |
|  | Motor scale | .011 | -.073 .096 | .794 |  | .008 | | -.070 .088 | .826 |  |  |

Note: n is presented for at-risk category, low risk category

At risk category for: stunting is <-2SD, SES is below mean SES for the sample, maternal literacy is inability to read and write and child gender is female.
